# RLIP76 Regulates PI3K/Akt Signaling and Chemo-Radiotherapy Resistance in Pancreatic Cancer

**DOI:** 10.1371/journal.pone.0034582

**Published:** 2012-04-03

**Authors:** Kathryn Leake, Jyotsana Singhal, Lokesh Dalasanur Nagaprashantha, Sanjay Awasthi, Sharad S. Singhal

**Affiliations:** Department of Diabetes and Metabolic Diseases Research, Beckman Research Institute, City of Hope, Comprehensive Cancer Center, Duarte, California, United States of America; Vanderbilt University Medical Center, United States of America

## Abstract

**Purpose:**

Pancreatic cancer is an aggressive malignancy with characteristic metastatic course of disease and resistance to conventional chemo-radiotherapy. RLIP76 is a multi-functional cell membrane protein that functions as a major mercapturic acid pathway transporter as well as key regulator of receptor-ligand complexes. In this regard, we investigated the significance of targeting RLIP76 on PI3K/Akt pathway and mechanisms regulating response to chemo-radiotherapy.

**Research Design and Methods:**

Cell survival was assessed by MTT and colony forming assays. Cellular levels of proteins and phosphorylation was determined by Western blot analyses. The impact on apoptosis was determined by TUNEL assay. The anti-cancer effects of RLIP76 targeted interventions *in vivo* were determined using mice xenograft model of the pancreatic cancer. The regulation of doxorubicin transport and radiation sensitivity were determined by transport studies and colony forming assays, respectively.

**Results:**

Our current studies reveal an encompassing model for the role of RLIP76 in regulating the levels of fundamental proteins like PI3K, Akt, E-cadherin, CDK4, Bcl2 and PCNA which are of specific importance in the signal transduction from critical upstream signaling cascades that determine the proliferation, apoptosis and differentiation of pancreatic cancer cells. RLIP76 depletion also caused marked and sustained regression of established human BxPC-3 pancreatic cancer tumors in nude mouse xenograft model. RLIP76 turned out to be a major regulator of drug transport along with contributing to the radiation resistance in pancreatic cancer.

**Conclusions/Significance:**

RLIP76 represents a mechanistically significant target for developing effective interventions in aggressive and refractory pancreatic cancers.

## Introduction

Pancreatic cancer is the fourth leading cause of cancer-related deaths among men and women [1]. Approximately 95% of malignant tumors within the pancreas arise from the exocrine tissue. Among pancreatic exocrine malignancies, 80% to 90% are ductal adenocarcinomas [2], [3]. Fewer than 20% of patients with pancreatic cancer have disease that is macroscopically confined to the pancreas at diagnosis with the rest of the patients presenting with locally advanced and distant visceral metastases, usually involving the liver [4]. Pancreatic cancers possess multiple aberrations in the cellular signaling cascades and are characteristically known for their invasive phenotype and refractoriness to conventional modes of therapy. The treatment of pancreatic cancer is frequently met with disappointing outcomes due to the development of resistance to therapy consequent to activation of a number of survival promoting proteins which transduce signals from extracellular signaling molecules such as epidermal growth factor (EGF), transforming growth factor (TGF), or insulin-like growth factors (IGF1) [5], [6]. Molecular studies have also characterized the mutations of K-ras oncogene in 80% or more of ductal adenocarcinomas [7]. The PI3K/Akt pathway plays a significant role in signal transduction from upstream growth factor receptors as well as oncogenic K-ras [8]–[12]. PI3K/Akt signaling also represents a potent and fundamental axis of signal relay that determines the basal survival and resistance to the apoptotic effects of chemo-radiotherapy in a variety of cancers, which makes PI3K/Akt pathway a central focus of mechanistic investigations in pancreatic cancer [13], [14]. Currently, there is no effective treatment for pancreatic cancer and conventional chemo-radiotherapy has shown very limited success in improving patient survival. The overall survival rate of pancreatic cancer patients is ∼5%. Hence, the investigation of the mechanisms of action of novel targets which can regulate the molecular changes that drive the pancreatic cancer survival and refractoriness to therapy will facilitate the development of effective interventions for pancreatic cancer [4], [15].

Mercapturic acid pathway plays a critical role in regulating the cellular antioxidant potential and resistance to chemo-radiotherapy [16]. Glutathione (GSH) is a sulfur containing small molecule in the cells that is essential to protect the cells from multiple toxic stimuli that induce cell death [17]. During the first step of mercapturic acid pathway, the cellular glutathione S-transferases (GSTs) catalyze the conjugation of administered chemotherapy drugs and products of lipid peroxidation, induced consequent to radiotherapy, with GSH to form glutathione-conjugates (GS-Es) [18]. The GS-Es are still toxic to the cells and need to be effluxed out of cells in order to protect the cells from cell death. During the second step of mercapturic acid pathway, the GS-Es are effluxed out of cells and this process is mediated by energy-dependent transport pumps present in the cell membranes [19]. In our extensive previously published studies, we have shown that RLIP76 is a primary mercapturic acid pathway transporter that removes GS-Es resulting from products of lipid peroxidation and chemotherapy drugs from the cells. This function of RLIP76 is more important for cancer cells as compared with normal cells as depletion of RLIP76 does not kill normal cells, but is very effective in killing cancer cells of nearly all types [20]–[24].

Our recently published studies indicate that RLIP76 is also a stress-responsive GS-E transporter required for clathrin-dependent endocytosis (CDE), which is required for regulation of receptor-ligand signaling at the cell membrane receptors [25]. In the context of striking chemo-radiotherapy resistance of pancreatic cancers and the fundamental role of RLIP76 as an important mercapturic acid pathway transporter that is essential for survival and therapy resistance in cancers, we investigated the role of RLIP76 in regulating the critical signaling proteins involved in relaying the inputs from multiple upstream survival pathways and mechanisms contributing to chemo-radiotherapy resistance in pancreatic cancer.

## Materials and Methods

### Materials

Doxorubicin (DOX, adriamycin) was obtained from Adria Laboratories (Columbus, OH). ^3^H-GSH (3,000 Ci/mmol) was purchased from Pharmacia Biotech (Piscataway, NJ). ^14^C-DOX (specific activity 44.8 Ci/mmol) was purchased from NEN Life Sciences (Boston, MA). Polyclonal rabbit-anti-human rec-RLIP76 IgG as well as pre-immune IgG were prepared and purified as described previously [26], [27]. MRP (N19; cat # sc7774), Pgp (C19; cat # sc1517), Akt (cat # Sc8312), GAPDH (cat # sc32233), Bcl2 (cat # sc509), Bim (cat # sc11425), and cyclin B1 (cat # sc595) antibodies were obtained from Santa Cruz Biotechnology (Columbus, OH). pAkt (S^473^; cat # 05-736) antibody was procured from Upstate Cell Signaling (Lake Placid, NY). Antibodies against PI3K (cat # 4292S), pPI3K (Y^458^; cat # 4228), and PCNA (cat # 2586S) were from Cell Signaling Technologies (Danvers, MA). E-cadherin (cat # C3621), β-actin (cat # A5441), and cdk4 (cat # DCS-35) antibodies were from Sigma-Aldrich Corp. (St. Louis, MO.) and Neomarkers (Fremont, CA), respectively. TUNEL fluorescence detection kit was purchased from Promega (Madison, WI). DNP-SG was synthesized from CDNB and GSH according to the method described by us previously [28]. All animal experiments were carried out in accordance with the Institutional Animal Care and Use Committee (IACUC) approved protocol. RLIP76 antisense was purchased from Biosynthesis, Inc., (Lewisville, TX) [22], and RLIP76 siRNA was purchased from Dharmacon Research (Lafayette, CO), as described previously [29].

### Animals

Hsd: Athymic nude nu/nu mice were obtained from Harlan, Indianapolis, IN. Animals were maintained at the Beckman Research Institute, City of Hope National Medical Center, Duarte, CA. All animal experiments were carried out in accordance with an approved protocol (# 11016) by Beckman Research Institute, City of Hope National Medical Center Institutional Animal Care and Use Committee (IACUC).

### Cell Lines and Cultures

Human umbilical vascular endothelial cells (HUVEC) were kindly provided by Dr. Fiemu Nwariaku, University of Texas Southwestern Medical Center, Dallas, TX, as described previously (22–24), and cultured at 37°C in a humidified atmosphere of 5% CO_2_ in EGM-2 bullet kit medium supplemented with 10% (v/v) heat-inactivated FBS and 1% (v/v) Pen-Strep (P/S) solution. Human pancreatic cancer (BxPC-3 and Panc-1) cell lines were purchased from American Type Culture Collection, Manassas, VA, and cultured at 37°C in a humidified atmosphere of 5% CO_2_ in MEBM and RPMI-1640 medium supplemented with 10% (v/v) heat-inactivated FBS, 1% (v/v), Pen-Strep (P/S) solution, 2 mM L-glutamine, 10 mM HEPES, 1 mM sodium pyruvate, 4.5 g/L glucose, and 1.5 g/L sodium bicarbonate. All cells were tested for *Mycoplasma* once every 3 months.

### MTT cell viability assay

Cell number/ml in an aliquot of cells growing in log phase was determined by counting trypan blue excluding cells in a hemocytometer and 20,000 cells were plated into each well of 96-well flat-bottomed micro-titer plates. After 12 h incubation at 37°C, medium containing either pre-immune IgG or anti-RLIP76 IgG (40 µg/ml final concentration) were added to the cells. After 24–48 h incubation, 20 µl of 5 mg/ml MTT was introduced to each well and incubated for 2 h of exposure. The plates were centrifuged and medium was decanted. Cells were subsequently dissolved in 100 µl DMSO with gentle shaking for 2 h at room temperature, followed by measurement of OD_570 _nm [30]. Eight replicate wells were used in each point in each of three separate measurements. Measured absorbance values were directly linked with a spreadsheet for calculation of IC_50_, defined as the drug concentration that reduced formazan formation by 50%. Depletion of RLIP76 expression in cells by RLIP76 siRNA and RLIP76 antisense were measured as follows: cells were incubated for 3 h with 0–2 µg/well of either RLIP76 siRNA using Transmessenger Transfection Reagent (Qiagen) or RLIP76 anti-sense using Maxfect transfection reagent (MoleculA) according to the manufacturer provided protocols.

### Cloning, prokaryotic expression, and purification of RLIP76

Purified RLIP76 protein (1965 bp; 655 aa) was obtained from *E. coli* BL21(DE3) expressing the pET30a(+) plasmid containing full-length cDNA corresponding to the sequence of RLIP76. The purification was carried out using DNPSG-affinity resin as described previously and purity was confirmed by Western blot analyses [26], [31].

### Functional reconstitution of purified rec-RLIP76 into artificial liposomes and transport studies

Purified RLIP76 was dialyzed against reconstitution buffer (10 mM Tris-HCl, pH 7.4, 2 mM MgCl_2_, 1 mM EGTA, 100 mM KCl, 40 mM sucrose, 2.8 mM BME, 0.05 mM BHT, and 0.025% polidocanol). An aqueous emulsion of soybean asolectin (40 mg/ml) and cholesterol (10 mg/ml) was prepared in the reconstitution buffer by sonication and 0.1 ml of this mixture was added to 0.9 ml aliquot of dialyzed and purified rec-RLIP76 protein. The reaction mixture was sonicated at 50 W for 30 sec. Vesiculation was initiated by the addition of 200 mg SM-2 Bio-beads pre-equilibrated in the reconstitution buffer without polidocanol. Vesiculation was carried out for 4 h at 4°C, the SM-2 Bio-beads were removed by centrifugation and the vesicles (RLIP76-liposomes) were collected. The collected fraction yields primarily unilammelar vesicles with median diameter of 0.25 µm and intravesicular/extravesicular volume ratio of 18 µL/mL. Control vesicles (control-liposomes) were prepared using an equal amount of albumin or crude protein from *E. coli* not expressing RLIP76. ATP-dependent transport of ^14^C-DOX and ^3^H-DNPSG in the rec-RLIP76 reconstituted proteoliposomes was performed by rapid filtration technique using the exact protocol described by us previously where the efficiency of delivery for proteoliposomes has been established [26].

### Preparations of crude membrane fractions for Western blot analyses

Crude membrane fractions were prepared from the normal and cancer cell lines using established procedures as described previously [22]. Briefly, cells were pelleted and washed with balanced salt solution (138 mM NaCl, 5 mM KCl, 0.3 mM KH_2_PO_4_, 0.3 mM Na_2_HPO_4,_ 4 mM NaHCO_3_, and 5.6 mM glucose, pH 7.4) three times. Washed cells were lysed in 10 mM Tris-HCl, pH 7.4, containing 1.4 mM BME, 0.1 mM PMSF, 0.05 mM BHT, 0.1 mM EDTA and 0.5% (v/v) polidocanol. Lysates were sonicated three times for 30 sec at 50W and incubated for 4 h at 4°C with occasional shaking. After incubation, the resultant preparation was centrifuged at 100,000-× g for 60 min at 4°C. The supernatant was collected and subjected to SDS-PAGE. Levels of RLIP76 protein in normal and cancer cells was measured by Western blot and ELISA using anti-RLIP76 IgG as previously described [29], [31]. Purified rec-RLIP76 with purity assessed by amino acid composition analysis was used to generate calibration curves.

### Transport studies in IOVs

Crude membrane vesicles (inside-out vesicles, IOV) were prepared from the normal (HUVEC) and malignant (BxPC-3 and Panc-1) cell lines using established procedures as described by us for the K562 cells [26]. Transport studies of DOX and DNP-SG in IOVs were performed by the method as described previously [26]. IOVs were separately coated with 40 µg/ml final concentration of either anti-RLIP76 IgG, anti-MRP1 IgG, or anti-Pgp IgG and used to measure the ATP-dependent uptake of ^14^C-DOX. ATP-dependent uptake of ^14^C-DOX was determined by subtracting the radio-activity (cpm) of the controls without ATP from that of the experimental groups containing ATP. The transport of DOX was calculated in terms of pmol/min/mg IOV protein. The transport of ^3^H-DNP-SG was measured in a similar manner.

### Tumor xenografts model

Hsd: Athymic nude nu/nu mice were obtained from Harlan, Indianapolis, IN. All animal experiments were carried out in accordance with a protocol approved by the Institutional Animal Care and Use Committee (IACUC). Thirty-six 10-weeks-old mice were divided into six groups of 6 animals (treated with pre-immune serum, scrambled siRNA, scrambled antisense, RLIP76 antibodies, RLIP76 siRNA and RLIP76 antisense DNA). All 36 animals were injected with 2×10^6^ human pancreatic cancer cells (BxPC-3) suspensions in 100 µl of PBS, subcutaneously. Animals were examined daily for signs of tumor growth. Treatment was administered when the tumor surface area exceeded ∼42 mm^2^ (day 47 after tumor cell injection, i.e. day 1 for treatment). Treatment consisted of 200 µg of either RLIP76 antibodies, RLIP76 siRNA or RLIP76 antisense in 100 µl PBS. Control groups were treated with 200 µg/100 µl pre-immune serum, scrambled siRNA and scrambled antisense. Tumors were measured in two dimensions by calipers.

### Colony formation assay

Cells (0.1×10^6^ cells/500 µL) were incubated with scrambled antisense and RLIP76 antisense (10 µg/ml final concentration) for 24 h. After 24 h, aliquots of 50 and 100 µL of were further incubated in 60 mm size Petri dishes, separately, in a total volume of 4 ml medium to in each Petri dish. For irradiation experiments, control and RLIP76-proteoliposomes (50 µg/ml final concentration, 24 h) treated cells were irradiated at 100, 200, 500 and 1000 cGY using Varian Clinac 2100C Linear Accelerator with 6 MeV photon beam. After 7 days, adherent colonies were fixed, stained with 0.5% methylene blue for 30 min., and colonies were counted using Innotech Alpha Imager HP [32].

### Effect of RLIP76 antisense on apoptosis by TUNEL assay

Cells (∼1×10^6^) were grown on the cover slips and treated with scrambled antisense and RLIP76 antisense (10 µg/ml final concentration) in Maxfect transfection reagent (MoleculA). Apoptosis was determined by the labeling of DNA fragments with terminal deoxynucleotidyl-transferase dUTP nick-end labeling (TUNEL) assay using Promega apoptosis detection system according to the protocol provided by manufacturer [33].

### Statistical Analysis

All data were evaluated with a two-tailed unpaired student's t test or compared by one-way ANOVA and are expressed as the mean ± SD. For *in vivo* studies, drug-treatment values were compared with the control vehicle-treatment values. A *p* value<0.05 was considered statistically significant.

## Results

### Expression of RLIP76 in pancreatic cancer cells

**Figure 1:**
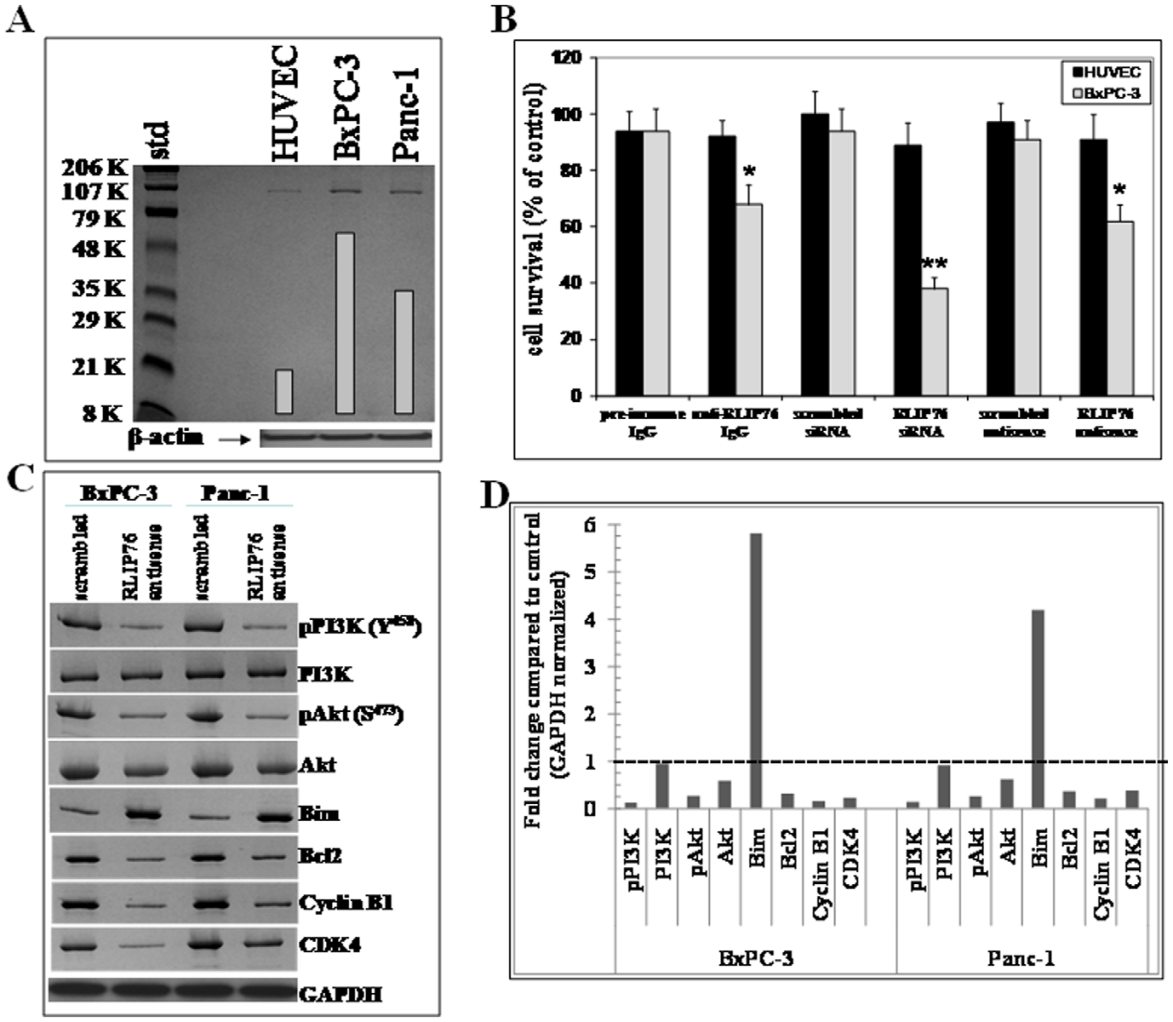
Comparison of RLIP76 levels in pancreatic cancer cells *vs* non-malignant cells. Aliquots of crude membrane fractions of pancreatic cancer cells (BxPC-3 and Panc-1) and normal control cells (HUVEC) containing 100 µg protein were used for SDS-PAGE and Western blotting. Intensity of the full-length RLIP76 protein (∼95 kDa) band was quantified by scanning densitometry using Innotech Alpha Imager HP. β-actin was used as an internal control (**panel A**). Impact of anti-RLIP76 IgG, RLIP76 siRNA and RLIP76 antisense on normal and pancreatic cancer cells: Effect of anti-RLIP76 IgG (40 µg/ml final concentration) on the cell survival was determined by MTT assay [29], [30]. Depletion of RLIP76 expression by RLIP76 siRNA and RLIP76 antisense (each 10 µg/ml final concentration) was done, using Transmessenger Transfection-Reagent-kit (Qiagen), and Maxfect Transfection-Reagent (Molecula, Inc.), respectively, according to the manufacturer's instructions. Cell survival was measured by MTT cytotoxicity assay 48 h after treatment. The values are presented as mean ± SD from two separate determinations with eight-replicates each (n = 16), **black bars**, normal HUVEC cells; **gray bars**, BxPC-3 pancreatic cancer cells (**panel B**) * p<0.05, ** p<0.01 compared to respective controls. Effect of RLIP76 antisense on PI3K and Akt signaling in pancreatic cancer cells: RLIP76 antisense caused inhibition of PI3K/Akt pathway in BxPC-3 and Panc-1 cells. Cells were treated with 10 µg/ml of RLIP76 antisense for 24 h and immune-blotted for pPI3K, PI3K, pAkt, Akt, Bim, Bcl2, cyclin B1 and CDK4. The same blot was stripped and reprobed for GAPDH to ensure equal protein loading (**panel C**). Bar diagram shows the quantitation of respective Western blots. Dotted line represents no significant change as observed with scrambled antisense (**panel D**).

Western blot analyses of the membrane protein extracts from human normal and pancreatic cancer cells indicated the presence of relatively larger amounts of RLIP76 in pancreatic cancer cells compared to normal cells (**Fig. 1A**). Following the characterization of enhanced expression of RLIP76, we further investigated the effect of RLIP76 inhibition or depletion in pancreatic cancer cells. The results from assessing RLIP76 protein levels as presented in **Table 1** indicate the amounts of total crude membrane proteins obtained from 10^8^ cells in log-phase of growth. RLIP76 protein represented 12±2 µg/10^8^ normal cells and ∼40±3 µg/10^8^ pancreatic cancer cells, respectively (0.19% and ∼0.61% of the total detergent soluble protein from the membranes of normal and pancreatic cancer cells, respectively). The expression of RLIP76 in pancreatic cancer cells was in comparable range relative to results from various other cell lines in our previous studies [22], [24], [29].

**Table 1:** RLIP76 protein expression in human normal and pancreatic cancer cells.

	Total crude protein	RLIP76 protein	
	(mg/10^8^ cells)		
		µg/10^8^ cells	% of total
			crude protein
**Nonmalignant**			
HUVEC (umbilical endothelial)	6.28±0.58	12±2	0.19
**Malignant**			
BxPC-3	6.74±0.72	42±3	0.62
Panc-1	6.46±0.64	38±3	0.59

Cell lines were cultured in respective medium and homogenate was prepared from 10^8^ cells. RLIP76 was purified from total crude membrane fraction using DNP-SG affinity column chromatography and quantified by ELISA [26], [29], [31]. Values represent mean ± s.d. from three separate determinations.

### RLIP76 inhibition or depletion causes cytotoxicity in pancreatic cancer cells

The initial cytotoxic effects of RLIP76 inhibition in pancreatic cancer cells were assessed by RLIP76 inhibition using anti-RLIP76 IgG and RLIP76 depletion using RLIP76 siRNA or RLIP76 phosphorothioate antisense by an established MTT cell survival assay [22], [30]. Protein-A affinity purified immunoglobulin fraction obtained from the pre-immune serum was used as control. Anti-RLIP76 IgG used in these experiments was previously shown by Ouchterlony double immuno-diffusion assay to be non-cross-reactive with any other proteins including Pgp or MRP [31]. Cells were treated with pre-immune IgG, scrambled siRNA, scrambled antisense, anti-RLIP76 IgG, RLIP76 siRNA or RLIP76 antisense for 24 h. The cytoxicity of all three RLIP76 targeting agents, RLIP76 siRNA, antibody and antisense, was preferentially directed towards the malignant cells as compared with the non-malignant HUVEC cells, which was similar to our observations with other malignant (lung, melanoma, kidney, prostate) and non-malignant cell lines [22], [24], [29], [34]. In contrast with the previous results seen with lung or colon cancers (in which all three modalities gave similar results), RLIP76 antisense was significantly more effective in killing pancreatic cancer cells than the RLIP76 antibody (**Fig. 1B**). RLIP76 mediates survival and proliferation of cancer cells by multiple mechanisms which include enhanced detoxification of products of lipid peroxidation as well as regulation of intracellular signaling pathways [19], [25], [35]. The enhanced efficacy of RLIP76 antisense was a striking finding which stimulated further detailed investigation of RLIP76 depletion in regulating the levels of critical intracellular proteins in pancreatic cancer cells.

### RLIP76 antisense down-regulates PI3K/Akt pathway in pancreatic cancer cells

BxPC-3 and Panc-1 cells were treated with RLIP76 antisense for 24 h followed by Western blot analyses to study the impact on critical intracellular proteins (**Figs. 1C and D**). PI3K/Akt is constitutively activated in majority of pancreatic tumors [11]. RLIP76 antisense treatment significantly suppressed the phosphorylation of PI3K, a common upstream signal signaling node which tranduces mitogenic signals consequent to cell membrane receptors like growth factors and integrins. RLIP76 antisense treatment also significantly reduced the protein levels and phosphorylation of Akt in both BxPC-3 and Panc-1 cells. Interestingly, phosphorylation of Akt and PI3K was not detected in normal HUVEC cells (data not shown), which is consistent with the general understanding that PI3K or Akt are activated in transformed cells [36]. The PI3K and Akt represent significant nodes of signal relay which in turn regulate the critical downstream targets that are involved in apoptosis, proliferation and maintaining the phenotype of cancers. The activation of Akt leads to repression of the expression of E-cadherin [37]. E-cadherin is considered a marker of normal epithelial phenotype and loss of E-cadherin is associated with “Epithelial mesenchymal transition (EMT)” and an aggressive phenotype in cancers [38]. The activation of PI3K leads to further activation of mTOR which suppresses the expression of pro-apoptotic Bim and favors survival and proliferation of cancer cells [39]. The enhanced levels of anti-apoptotic Bcl2 and cyclin dependent kinase 4 (CDK4) have been shown to mediate resistance to mTOR inhibition [40]. Depletion of RLIP76 by antisense significantly increased the levels of the pro-differentiation marker E-cadherin and pro-apoptotic protein Bim while decreasing the levels of anti-apoptotic protein Bcl2, cyclin B1 and CDK4 in both BxPC3 and Panc-1 pancreatic cancer cells. Thus, depletion of RLIP76 had a significant impact on critical mediators of PI3K/Akt signaling axis in pancreatic cancer (**Figs. 1C and D**).

### Effect of RLIP76 depletion on clonogenic survival and apoptosis

**Figure 2:**
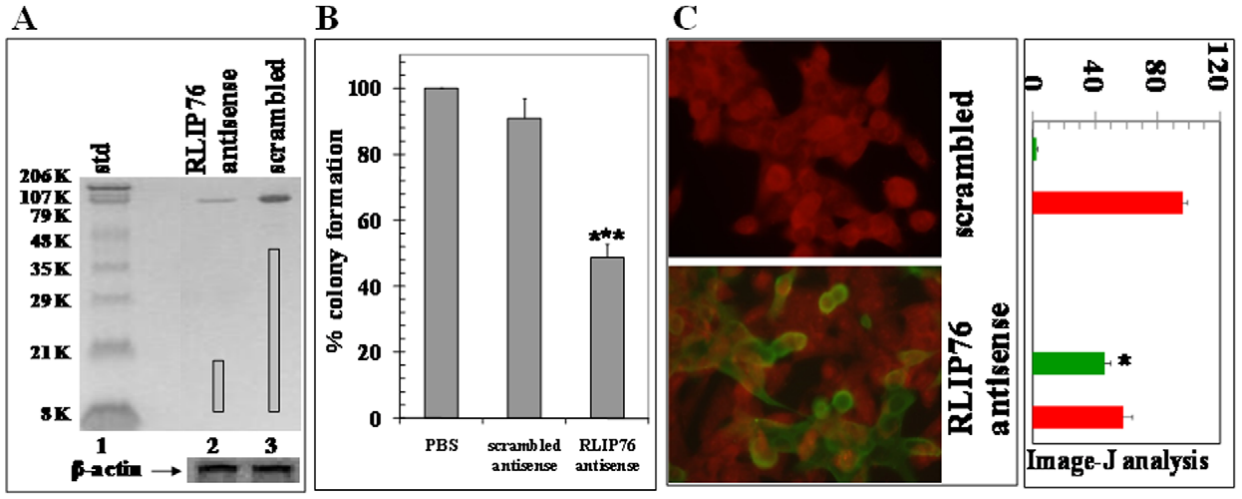
Effect of RLIP76 antisense on RLIP76 expression and apoptosis. Depletion of RLIP76 by RLIP76 antisense using Western blot analyses, lane 1 contained standard, lanes 2 and 3, contained crude membrane fraction of pancreatic cancer (BxPC-3) cells 24 h after treatment of RLIP76 antisense and scrambled antisense, respectively. Results were quantified by scanning densitometry using Innotech Alpha Imager HP. β-actin was used as an internal control (**panel A**). Colony-forming efficiency in BxPC-3 cells after treatment of antisense, was performed by staining the cells with methylene blue and the colonies were counted using Innotech Alpha Imager HP. Values are means ± S.D. of three separate experiments. *** p<0.005 compared with no or scrambled antisense treatment (**panel B**). Apoptosis in BxPC-3 cells by RLIP76 antisense by TUNEL assay, scrambled antisense (upper panel) and RLIP76 antisense (lower panel) treated cells. Apoptotic cells showed green fluorescence and characteristic of cell shrinkage. The adjacent bar diagram shows Image J analyses of TUNEL assay representing percentage of TUNEL positive apoptotic cells (green) and normal live cells (red). * p<0.05 (**panel C**).

We further confirmed the impact of RLIP76 depletion on the proliferative potential and cell survival by assessing the clonogenic potential and apoptosis in BxPC3 pancreatic cells. RLIP76 antisense treatment caused RLIP76 depletion as detected by Western blot (**Fig. 2A**) and inhibited the clonogenic potential as determined by colony-forming assay (**Fig. 2B**). The impact of RLIP76 on apoptosis was assessed by TUNEL assay. The results of the TUNEL assay showed no detectable apoptosis with scrambled antisense while RLIP76 antisense caused apoptosis in the BxPC-3 pancreatic cancer cells. Quantification of red and green fluorescence by image -J analysis confirmed the qualitative fluorescent findings that the RLIP76 depletion by antisense was effective in inducing apoptosis (**Fig. 2C**).

### RLIP76 depletion causes regression of pancreatic cancer xenografts in nude mice

**Figure 3:**
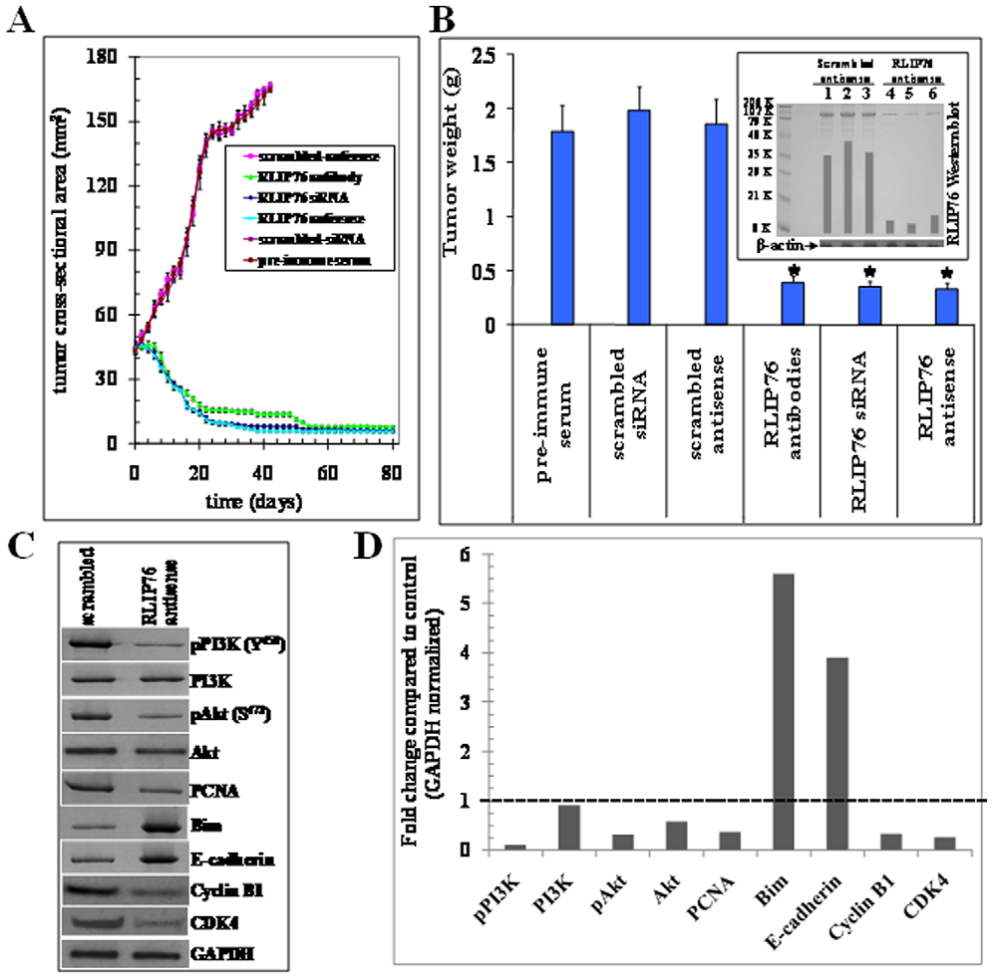
Treatment with RLIP76 antibody, siRNA or antisense causes regression of established BxPC-3 pancreatic cancer xenografts. For xenografts studies, we used thirty-six 11-weeks-old Hsd: Athymic nude nu/nu mice (Harlan, Indianapolis, IN) randomized 6 animals each into six groups as follow: 1) pre-immune serum, 2) scrambled siRNA, 3) scrambled anti-sense DNA, 4) RLIP76 antibodies, 5) RLIP76 siRNA and 6) RLIP76 antisense. All 36 animals were injected with 2×10^6^ human pancreatic cancer cells (BxPC-3) suspensions in 100 µl of PBS, subcutaneously into one flank of each nu/nu nude mouse. Animals were examined daily for signs of tumor growth and body weights. When tumors reached a cross-sectional area of ∼42 mm^2^ (47 d later), animals were randomized in to treatment groups as indicated in Figure 4. Treatment consisted of 200 µg of RLIP76 antibodies, siRNA or antisense in 100 µl PBS. Control groups were treated with 200 µg/100 µl of either pre-immune serum, scrambled siRNA or scrambled anti-sense DNA. Tumors were measured in two dimensions using calipers. Tumor measurements are presented with all control groups (pre-immune IgG, scrambled siRNA or antisense) versus all treated groups (anti-RLIP76 IgG, RLIP76 siRNA, or anti-sense) (**panel A**). Tumor-weight is reduced at day 47 after the treatment start (**panel B**) and tumor RLIP76 is depleted by antisense (**panel B, inset**). Tumors were excised 48 h after antisense-treatment, weighed, and homogenized, and aliquots containing 100 µg protein from each, were loaded to SDS-PAGE for Western blotting against anti-RLIP76 IgG (n = 3; lanes 1–3, scrambled antisense treated, and lanes 4–6, RLIP76-antisense treated). Results were quantified by scanning densitometry. Membrane was stripped and re-probed with β-actin antibody to verify equal protein loading, * p<0.05 compared with respective controls. Western blot analysis of tumor tissue lysates: Mechanistic basis for the tumor growth inhibition by RLIP76-antisense was determined in the tumor lysates from control and RLIP76-antisense treated mice. Tumor tissues were homogenized, and ∼70 µg protein was resolved on SDS-PAGE and probed for pPI3K, PI3K, pAkt, Akt, PCNA, Bim, E-cadherin, Cyclin B1 and CDK4. The blots were stripped and reprobed for GAPDH to ensure equal protein loading (**panel C**). Bar diagram represents densitometry analyses. Dotted line represents no significant change as observed with scrambled antisense (**panel D**).

**Figure 4:**
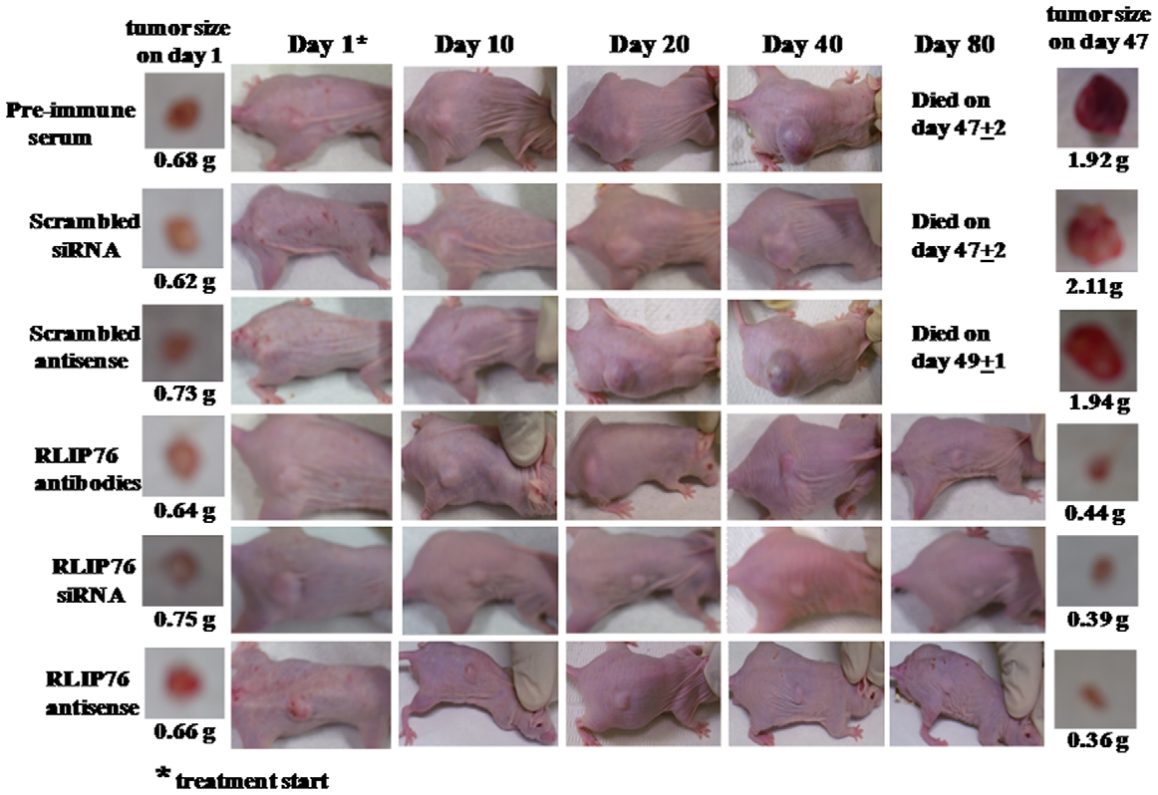
Effect of anti-RLIP76 IgG, RLIP76 siRNA and RLIP76 antisense on the size of subcutaneously implanted human pancreatic cancer cells (BxPC-3) in nude mice. Hsd: Athymic nude nu/nu mice were obtained from Harlan, Indianapolis, IN. All animal experiments were carried out in accordance with a protocol approved by the Institutional Animal Care and Use Committee (IACUC). Thirty-six 11-weeks-old mice were divided into six groups of 6 animals (treatment with pre-immune serum, scrambled siRNA, scrambled anti-sense DNA, RLIP76 antibodies, RLIP76 siRNA and RLIP76 antisense). All 36 animals were injected with 2×10^6^ human pancreatic cancer cells suspensions in 100 µl of PBS, subcutaneously into one flank of each nu/nu nude mouse. Animals were examined daily for signs of tumor growth. When tumors reached a cross-sectional area of ∼42 mm^2^ (47 days later), animals were randomized treatment groups as indicated in the figure. Treatment consisted of 200 µg of RLIP76 antibodies, siRNA or antisense in 100 µl PBS. Control groups were treated with 200 µg/100 µl pre-immune serum, scrambled siRNA or scrambled anti-sense DNA. Tumors were measured in two dimensions using calipers. Photographs of animals were taken at day 1, day 10, day 20, day 40 and day 80 after treatment are shown for all groups. Photographs of tumors were also taken at day 1 and day 47 after treatment.

The above *in vitro* observations reflecting the anti-neoplastic effects of RLIP76 depletion were further investigated *in vivo* mice xenograft model of pancreatic cancer. Tumor-bearing animals with established *s.c*. implanted BxPC3 pancreatic cell tumors (∼42 mm^2^) were treated with 200 µg of either RLIP76 antibody, RLIP76 siRNA or RLIP76 antisense by *i.p.* injection. Weight gain was comparable to non-tumor-bearing controls, and no overt-toxicity was evident. The treatment with RLIP76 antibody, RLIP76 siRNA or RLIP76 antisense resulted in rapid and dramatic reductions in tumors (**Figs. 3A and B**). The RLIP76 antibody, RLIP76 siRNA or RLIP76 antisense treated animals bearing established s.c. tumors were alive for ∼298 days without any evidence of recurrence. There was uncontrolled growth in all the control groups treated with pre-immune serum, scrambled siRNA and scrambled antisense and hence the control groups were censored by ∼49 days (**Fig. 4**). The efficacy of tumor-volume reduction was evident from tumor-weights at day 47 after the beginning of treatment (**Fig. 3B**). The effectiveness of RLIP76 antisense in depleting tumor RLIP76 *in vivo* is demonstrated in the Western blot for RLIP76 from tumor homogenates (**Fig. 3B** **, inset**). Following RLIP76 depletion and inhibition, regression of tumor implants were seen in all animals, without any overt toxic effects, or effects on weight gain.

### Tumor growth inhibition was associated with inhibition of PI3K/Akt pathway

Following *in vivo* studies, we further analyzed the levels of PI3K, Akt and critical downstream signal transducers in resected control and RLIP76 antisense treated pancreatic tumor tissue lysates by Western blot analyses (**Figs. 3C and D**). The phosphorylation of PI3K and Akt were drastically suppressed by RLIP76 antisense treatment. The expression level**s** of Akt, but not PI3K, was also reduced in the tumors of RLIP76 antisense-treated mice. Next, we investigated the levels of pro-apoptotic Bim, E-cadherin, cyclin B1, and CDK4, which are known to play a critical role in regulating the proliferation, differentiation and apoptosis of pancreatic tumors [11], [37]–[40]. We observed a striking decrease in the levels of PCNA, cyclin B1 and CDK4 levels and increase in the levels of tumor suppressor E-cadherin and pro-apoptotic protein Bim in the tumor tissue lysates of RLIP76 antisense-treated mice as compared with controls. These observations confirmed our *in vitro* findings that RLIP76 depletion induces potent anti-proliferative and pro-apoptotic effects in pancreatic cancers.

### Assessment of transport activity of RLIP76 in pancreatic cancer cells

Following the analysis of the role of RLIP76 in regulating pancreatic cancer survival and apoptosis *in vitro* and *in vivo*, we further investigated the significance of the transport function of RLIP76 in regulating therapeutic resistance in pancreatic cancer cells. We have previously shown that proteo-liposomes reconstituted with RLIP76 mediate ATP-dependent transport of doxorubicin (DOX) and other drugs [26]. For assessing the transport activity of RLIP76, we compared the ATP-dependent uptake of ^14^C-DOX and ^3^H-DNP-SG in crude membrane in-side-out vesicles (IOV) prepared separately from the membranes of control and pancreatic cancer cells (**Table 2**). Results from the measurements of ATP-dependent transport of ^14^C-DOX and ^3^H-DNPSG revealed greater transport of both substrates in pancreatic cancer cells reflecting a positive correlation between RLIP76 expression and transport-activity in pancreatic cancer cells which had higher levels of RLIP76 (**Table 1**) compared to normal cells [^14^C-DOX transport (pmol/min/mg protein) - A. Normal cells: 28±4; B. Pancreatic cancer cells: (i) BxPC3: 144±12 and (ii) Panc-1: 131±10. ^3^H-DNP-SG transport (pmol/min/mg)- A. Normal cells: 101±8; B. Pancreatic cancer cells: (i) BxPC3: 681±48 and (ii) Panc-1: 611±52] .

**Table 2:** RLIP76 transport activity in human normal and pancreatic cancer cells.

	Transport activity	
	(pmol/min/mg protein)	
	^14^C-DOX	^3^H-DNP-SG
**Nonmalignant**		
HUVEC (umbilical endothelial)	28±4	101±8
**Malignant**		
BxPC-3	144±12	681±48
Panc-1	131±10	611±52

For transport studies, in-side out vesicles (IOVs) prepared from 20×10^6^ cells was enriched for IOVs by wheat germ agglutinin affinity chromatography [26]. Transport activity was calculated from measurements of uptake of ^14^C-DOX (sp. activity, 8.5×10^4^ cpm/nmol) and ^3^H-DNP-SG (sp. activity, 3.6×10^3^ cpm/nmol) into the IOVs (20 µg/30 µl reaction mixture) in the absence or presence of 4 mM ATP as described [26]. Each transport experiment was done in triplicates in three separate experiments (n = 9).

### Relative contribution of RLIP76 towards DOX-transport

**Figure 5:**
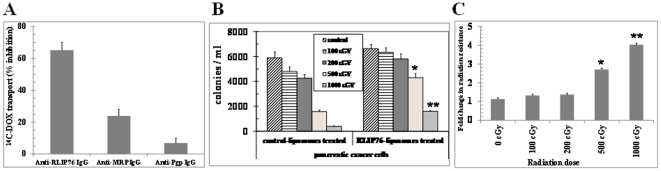
RLIP76 mediated DOX transport and radiation-protection in pancreatic cancer cells. The crude membrane vesicles (inside-out vesicles, IOV) from BxPC3 cells were separately coated with 40 µg/ml final concentration of either anti-RLIP76 IgG, anti-MRP1 IgG, or anti-Pgp IgG and used to measure the ATP-dependent uptake of ^14^C-DOX. ATP-dependent uptake of ^14^C-DOX was determined by subtracting the radio-activity (cpm) of the controls without ATP from that of the experimental groups containing ATP (**panel A**). Approximately, 2.5×10^3^ BxPC3 cells grown in RPMI-1640 medium, were treated with control and RLIP76-liposomes (50 µg/ml final concentration) for 24 h prior to radiation at 100, 200, 500 and 1000 cGY (6 MeV photons). After 7 days, cells were stained with methylene-blue and the colonies were counted using Alpha Imager HP [32], [33] (**panel B**) * p<0.05, ** p<0.01 compared to controls. The fold change in the radiation-resistance of RLIP76 *vs.* control-liposome treated pancreatic cancer cells at each dose of irradiation is represented in the bar diagram (**panel C**). The results presented are the mean and s.d. from three separate experiments (n = 9) * p<0.05, ** p<0.01 compared to control.

DOX is a common allocrite transported by RLIP76 [26], MRP1 [41], [42] and MDR1 [43], [44]. Hence, we further quantified the relative contribution of these transporters in the ATP-dependent transport of ^14^C-DOX in BxPC-3 cells using an immunological approach. We have previously shown that anti-RLIP76 IgG inhibit DOX-transport in IOVs [31]. Likewise, specific antibodies against MRP and Pgp also inhibit transport-activity in IOVs [45], [46]. We designed experiments in which IOVs prepared from BxPC-3 pancreatic cancer cells and separately coated with anti-RLIP76 IgG, anti-MRP1 IgG, or anti-Pgp IgG were used to measure the ATP-dependent uptake of ^14^C-DOX. Based on initial titration experiments, we used 40 µg/ml of each of the antibodies to inhibit the transport activity of their respective antigen present in the IOVs used in these experiments. In control vesicles coated with pre-immune IgG, the transport activity remained unaffected. Anti-RLIP76 IgG, which recognized only RLIP76 in crude extracts of BxPC-3 cells, inhibited 65±5% of total DOX-transport in IOVs prepared from BxPC-3 cells. Anti-MRP IgG also inhibited DOX-transport in a saturable manner, but the inhibition was less (24±4%) as compared to that observed with anti-RLIP76 IgG. Anti-Pgp IgG had a small but detectable inhibitory effect on DOX-transport (7±3%) (**Fig. 5A**). Importantly, these results established that RLIP76 accounts for a major two-third portion of the ATP-dependent transport of DOX in pancreatic cancer cells.

### Impact of RLIP76 on Radiation-Sensitivity

In our previous studies, we have shown that RLIP76 regulates the resistance to chemo-radiotherapy by ATP-dependent transport of products of lipid peroxidation and administered chemotherapy drugs [19]–[21], [47]. In this regard, we specifically sought to determine the role of RLIP76 in regulating the levels of radiation-sensitivity in pancreatic cancer cells. We first determined the X-irradiation sensitivity of the RLIP76 and control liposome treated BxPC3 human pancreatic cancer cells in dose-response studies utilizing 100–1000 cGY single dose X-irradiation, followed by colony-forming assays. In these studies, pancreatic cancer cells were loaded with RLIP76 by incubating with RLIP76-liposomes. Cells pretreated with RLIP76-liposomes were least sensitive to radiation. At each dose of radiation, the survival was significantly more when the cells were pretreated with RLIP76-liposomes before radiation exposure (**Fig. 5B**). Interestingly, RLIP76 supplementation by liposomes increased the radiation-resistance to a significantly higher level at higher doses of radiation compared to low doses of radiation. This finding reinforces that RLIP76 is essential to particularly survive oxidative stress induced by higher levels of radiation (**Fig. 5C**). Thus, the physiological significance of RLIP76 mediated transport of endogenously generated GS-Es is further indicated by these results showing that RLIP76 enriched cells are resistant to radiation toxicity.

## Discussion

**Figure 6:**
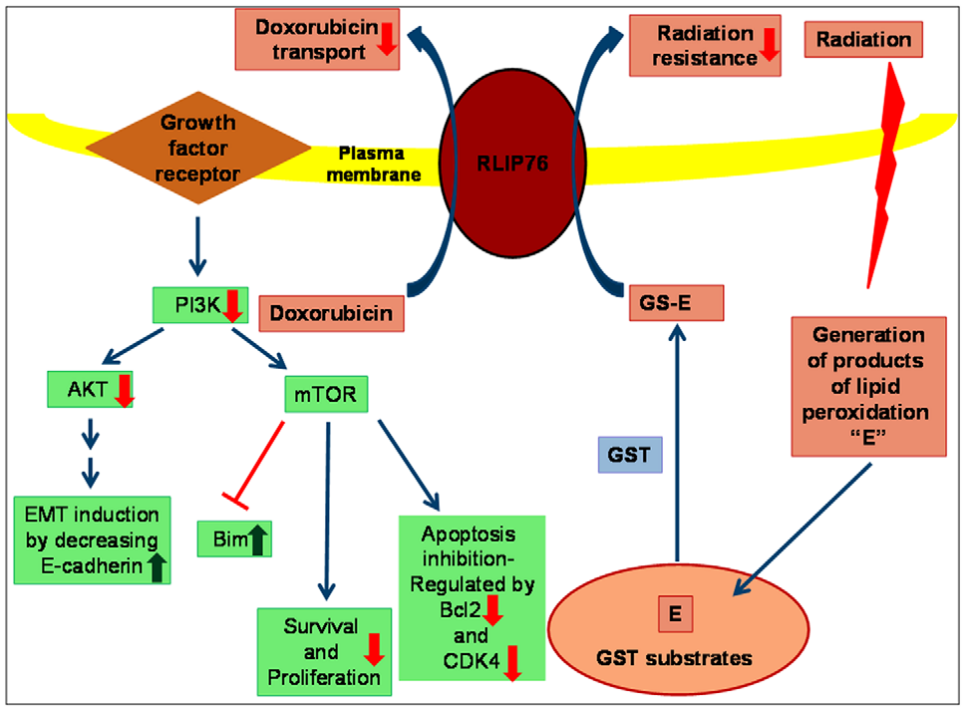
Major mechanisms contributing to RLIP76-mediated anti-cancer effects and chemo-radiotherapy resistance in pancreatic cancer. RLIP76 depletion leads to predominant inhibition of PI3K/Akt pathway as revealed by the inhibition of PI3K as well as decrease in the levels and phosphorylation of Akt. The associated downstream proteins that regulate differentiation, proliferation and apoptosis like E-cadherin, Bim, Bcl2 and CDK4 are also differentially regulated due to RLIP76 targeted interventions. The collective impact of RLIP76 depletion on the regulation of cellular signaling pathways as well as detoxification of glutathione-conjugates (GS-Es) of lipid peroxidation and chemotherapy drugs represents precious opportunities for the development of novel and effective therapeutic interventions for pancreatic cancers. *Green arrow*: Up regulation following RLIP76 depletion; *Red arrow*: down regulation following RLIP76 depletion; *Blue arrow*: Normal signal transduction.

RLIP76 is a major glutathione-conjugate transporter frequently over-expressed in cancer cells, and is strongly linked with resistance to apoptotic effects of a very wide variety of chemical as well as radiation induced oxidative stress [19]–[26]. In the current studies, we characterized that RLIP76 protein expression and specific activity for transport are relatively high in pancreatic cancer cells than non-malignant cells. Our present studies focused on elucidation of the role of RLIP76 in the regulation of critical survival signaling pathways prominently active in therapy resistant pancreatic cancers. Targeting RLIP76 is also a quite unique strategy because to date, there are no other targeting modalities that show such a broad spectrum of activity (lung, colon, prostate, kidney, melanoma, neuroblastoma, and pancreas) in both cell cultures and animal models, with inherent cancer specificity [22]–[24], [34], [48]. The significant feature of the present study was that depletion of RLIP76 effectively targeted the activation of PI3K/Akt pathway. Loss of E-cadherin alters the natures of cell contacts and given the highly metastatic and aggressive nature of pancreatic cancer, the over-expression of E-cadherin following RLIP76 depletion is a salient factor as it is a marker which distinguishes noncohesive pancreatic cancers from cohesive pancreatic cancers [49], [50]. Also, the decreased activation of PI3K, Akt and attenuated expression of Akt, anti-apoptoticBcl2, CDK4 along with a parallel increase in pro-apoptotic Bim provide a collective rationale for the effective regulation of PI3K/Akt pathway following RLIP76 depletion (**Fig. 6**).

RLIP76 serves as a functional nexus between glutathione mediated defense mechanisms and predominant survival signaling pathways in pancreatic cancer. RLIP76 represents a unique target in cancer therapy because it functions as the rate regulatory step in both mercapturic acid pathway as well as endocytosis [16], [19], [25]. The former is crucial for the protection of cells from stressors including oxidation, chemical toxins, and radiation. The later serves as a master regulator of receptor-ligand signaling. RLIP76 depletion by antisense has been shown to be safe in animal studies and current studies reveal the potential of RLIP76 targeted therapy to cause effective regression of pancreatic cancer. The applications of knowledge gained from present studies could include the optimization of combinations of other developing drugs for targeted therapy and improving the efficacy of available targeting agents.

The effective targets for cancer therapy must be desirably expressed differentially in particular cancers as compared with normal cells. Also, there should be an essential dependence of cancer cells on the target compared to non-malignant cells. Ideally, the target should be understood in the context of existing biochemical and signaling frameworks known to play a direct role in a particular carcinogenesis or in regulating the response to therapy. Results from our present studies provide strong support for the role of enhanced expression and function of RLIP76 in pancreatic cancers compared to normal cells. Thus, this study also lays a strong foundation for further clinical studies on the role of RLIP76 in pancreatic cancers. In conclusion, these findings from the present study regarding the role of RLIP76 in survival and chemo-radiotherapy resistance of pancreatic cancer can potentially impact the translational research for the development of more effective and targeted therapeutic strategies for pancreatic cancer.
